# The Public Health*Read at the meeting of the State Association of Health Officers, March 21, 1901.

**Published:** 1901-10

**Authors:** J. S. Brownlee

**Affiliations:** County Health Officer, Burnet, Texas


For Texas Medical Journal.

### The Public Health.*

BY J. S. BROWNLEE, M. D., COUNTY HEALTH OFFICER, BURNET, TEXAS.

"No creed without pathos will ever justify the great human hope, or conquer the great human heart."

The Great Physician and Savior of the race of man proved the truth of the tragic requirement in appealing to the "great human heart." Eternal life is the "great human hope." As we dwell upon this thought let us quote a table from a recent publication. Bones may endure 4000 years; lungs, 1500 years; skin, 1000 years; stomach, heart and liver, each 300 years, or more; and kidneys, 200 years, or more. If this is correct, the abuses of life are extreme and cry to intelligence for redress. In the first place, food and drink rank, essentially, graded according to quality not quantity. Water is a most important factor, the need of it being paramount, both as to quality and quantity. Water is seldom, when taken from the usual channels, fit for consumption, being compounded with earthy substances, and other poisonous materials. Especially is this so during a season of mortality, when the absorption from the air is aggravated.

Longevity is due to proper living, when not limited by hereditary taint. It is safe to say that every household can better its condition by more careful consideration of its water and food supply. The ideal home does not consider external as vital, above internal arrangements. Life and its sustenance are holy, and in this age of enlightenment should receive greater respect. It is regarding the importation of certain foods and fabrics that one can not expend too much thought.

The importance of sanitation has been discussed fully by able men, and every child should be a past master in the application of it to himself. Each individual should be a law unto himself, keeping, as the basis of gentility, his person immaculate, his diet per-

---

*Read at the meeting of the State Association of Health Officers, March 21, 1901.

fectly wholesome, and his premises in harmony with himself. Life, then, would be a grand harmony thrilled with sacred beauty. The pathos of the pilgrimage of progress lies in the graves of martyrs to undeveloped science—to unknown and unpracticed methods for the suppression of disease, as well as the ignorance regarding true living. Also, one might say, to the dormant principles of conscience sleeping at the gate of office. Controlled by public sentiment, the health official often hesitates to antagonize it. This applies to the interference of business, schools and social exchanges. But what are these without health? Man subordinates other expressions of nature in accordance with God's laws; and as these, too, were created according to his demands they are to be used intelligently by him. In the impoverished condition of a great empire, history tells us how every meal was regulated by law and measure. Even the great Josephine of France, when dining out, carried her loaf with her. This proves that necessity will find a means for the radification of the abnormal conditions of living—great excesses to be met by extreme frugality.

One of the first ways in which man showed his superiority over beasts was in the use of fire. Mythology records the contention of the gods over its use. Many of us, living in the country, have demonstrated its use when applied to sanitation. A very good rule is to burn or bury every discarded article, old shoes, old clothing, decaying vegetation; in fact, all perishable material. So closely are cleanliness and godliness allied that white is a symbol of purity, and enters largely into our ideas of holiness. But with our expansion of territory; with our liberal laws inviting immigration; with our loose municipal government; with our public institutions for the bringing together the great human family, there is need of courageous insistance upon official duty and individual responsibility.

Returning to the two powerful elements in life—fire and water—there is reason to think of the atmospheric impurities usually neglected in rural towns. The streams are usual dumping grounds for the scavenger; or, perhaps, the hillside just beyond the incorporation is used instead. With strong winds, the exhalations travel great distances. Not only this, but in our high and dry climate, the streams are dry eight months out of the year, and all the refuse cast therein becomes a sickening feast for ravenous birds—scavengers of the air. We endanger life for man and beast by such carelessness.

It seems that some cleanlier plan could be adopted. Instead of merely removing debris and refuse from the corporate limits, a furnace could be substituted, situate upon some isolated hill, or dug into it. Besides infecting the air, disease can be carried by insects; flies and mosquitoes being tested and found agents for depositing disease germs Rats, too, are very effective in carrying plague; they frequently devour their own dead. Drainage is very unsatisfactorily considered in country towns. Frequently farm houses are built with less consideration for the welfare of the inmates than is bestowed upon the stock yards. The one controlling influence is greed. It drives us in the country as well as in the city.

Every lofty and noble thing appeals to the "great human heart," and heroism will awaken such wild enthusiasm that the rocks almost quiver from its expression. The South is peculiarly expressive and responds to any nobility with a warm heart. Why, then, can not the desire for godly cleanliness and purity of bodily, mental and social growth find some zealot willing to endure the criticism of the public until the results of work can be seen, knowing that recognition must surely follow? Even the country doctor, acting as health officer, can do some work of the kind.

Public places of amusement, schools, the great highways of traffic and travel should be closed against the spread of an epidemic that is fatal. War and pestilence are close allies. Whether it be "Cuban itch" or smallpox, yellow fever or influenza, matters little regarding the conditions necessary for elimination.

During the Hundred Years' War (1336-1453) a terrible scourge, called "Black Death" (owing to the black spots upon the bodies of victims), desolated all Europe. In Germany over 1,000,000 were swept away, while about one-half the population of England fell victims. It was looked upon as a visitation from heaven, and a frenzy seized the people. Our children learn these things at the high schools, but will doubtless grow up with ideas uninfluenced by them. What would our great nation become should a war with China change our noble youths and beautiful children into lepers? While these thoughts are not meant as resentment, they are natural anticipations of the thinking man, with the bodily perfection, the moral elevation and the mental development of man at heart. It takes the pathos of a Christian creed to justify the great human hope, the pathos of crucification; where the evolution of mankind is accomplished through suffering unto death.

Death has stalked abroad, spreading his great hand to clutch our great as well as common man. To deter him is the wish of all. We can do so by using properly God's gifts.

